# OmniSARS2: A Highly Sensitive and Specific RT-qPCR-Based COVID-19 Diagnostic Method Designed to Withstand SARS-CoV-2 Lineage Evolution

**DOI:** 10.3390/biomedicines9101314

**Published:** 2021-09-26

**Authors:** Eduarda Carvalho-Correia, Carla Calçada, Fernando Branca, Nuria Estévez-Gómez, Loretta De Chiara, Nair Varela, Pilar Gallego-García, David Posada, Hugo Sousa, João Sousa, Maria Isabel Veiga, Nuno S. Osório

**Affiliations:** 1 Life and Health Sciences Research Institute (ICVS), School of Medicine, University of Minho, Campus Gualtar, 4710-057 Braga, Portugal; eduardaicorreia@med.uminho.pt (E.C.-C.); id6520@alunos.uminho.pt (C.C.); jcsousa@med.uminho.pt (J.S.); 2 ICVS/3B’s—PT Government Associate Laboratory, 4806-909 Guimarães, Portugal; 3 Center for Neuroscience and Cell Biology (CNC), University of Coimbra, 3004-504 Coimbra, Portugal; 4 Hospital de Braga, 4710-243 Braga, Portugal; fernando.branca@hb.min-saude.pt; 5 CINBIO, Universidade de Vigo, 36310 Vigo, Spain; nuestevez@uvigo.es (N.E.-G.); ldechiara@uvigo.es (L.D.C.); nairvarelarouco@gmail.com (N.V.); gpilargg@gmail.com (P.G.-G.); dposada@uvigo.es (D.P.); 6 Galicia Sur Health Research Institute (IIS Galicia Sur), SERGAS-UVIGO, 36213 Vigo, Spain; 7 Department of Biochemistry, Genetics, and Immunology, Universidade de Vigo, 36310 Vigo, Spain; 8 Virology Service and Molecular Oncology and Viral Pathology Group (CI-IPOP), Portuguese Oncology Institute of Porto (IPO Porto), 4200-072 Porto, Portugal; hugo.sousa@ipoporto.min-saude.pt

**Keywords:** SARS-CoV-2, COVID-19, RT-qPCR, B.1.1.7

## Abstract

Extensive transmission of SARS-CoV-2 during the COVID-19 pandemic allowed the generation of thousands of mutations within its genome. While several of these become rare, others largely increase in prevalence, potentially jeopardizing the sensitivity of PCR-based diagnostics. Taking advantage of SARS-CoV-2 genomic knowledge, we designed a one-step probe-based multiplex RT-qPCR (OmniSARS2) to simultaneously detect short fragments of the SARS-CoV-2 genome in ORF1ab, E gene and S gene. Comparative genomics of the most common SARS-CoV-2 lineages, other human betacoronavirus and alphacoronavirus, was the basis for this design, targeting both highly conserved regions across SARS-CoV-2 lineages and variable or absent in other *Coronaviridae* viruses. The highest analytical sensitivity of this method for SARS-CoV-2 detection was 94.2 copies/mL at 95% detection probability (~1 copy per total reaction volume) for the S gene assay, matching the most sensitive available methods. In vitro specificity tests, performed using reference strains, showed no cross-reactivity with other human coronavirus or common pathogens. The method was compared with commercially available methods and detected the virus in clinical samples encompassing different SARS-CoV-2 lineages, including B.1, B.1.1, B.1.177 or B.1.1.7 and rarer lineages. OmniSARS2 revealed a sensitive and specific viral detection method that is less likely to be affected by lineage evolution oligonucleotide–sample mismatch, of relevance to ensure the accuracy of COVID-19 molecular diagnostic methods.

## 1. Introduction

The coronavirus disease 2019 (COVID-19) is caused by a positive single-stranded RNA virus from the *Coronaviridae* family, named Severe Acute Respiratory Syndrome Coronavirus 2 (SARS-CoV-2). The detection of SARS-CoV-2 nucleic acids by reverse transcription (RT) and quantitative polymerase chain reaction (RT-qPCR) is the current gold standard COVID-19 diagnostic method. This virus transposed the species barrier and initiated human-to-human transmission in late 2019. In the beginning of the COVID-19 pandemic the design of the RT-qPCR oligonucleotides used was possible thanks to the rapid sequencing and availability of the first SARS-CoV-2 genome [1,2]. However, during the COVID-19 pandemic there was extensive global transmission of SARS-CoV-2 leading to thousands of spontaneous mutations in the viral genomes. As expected, some of these mutations become rare while others (with neutral or advantageous consequences for the virus) reached high frequencies in the population. This raised a diagnostic challenge requiring the redesign of the oligonucleotide sequences used in RT-qPCR assays to circumvent the oligonucleotide–sample mismatches caused by the mutations. Mismatches at the last five nucleotides of the 3′ ends of the primer oligonucleotides are considered of special relevance in impacting the efficiency of PCR. Furthermore, every mismatch, regardless of the nucleotide or its position in the primer or template, could decrease the thermal stability of the oligonucleotide–template duplex. This phenomenon has the potential to affect polymerization efficiency resulting in biased RT-qPCR results or reaction failure. In fact, the detrimental effect of primer–template mismatches is the foundation allowing PCR-based diagnostics to specifically distinguish closely related pathogens [3].

The remarkable genome sequencing effort performed by the international research community provides the information to address the primer–sample mismatch problem. We previously analyzed all high-quality SARS-CoV-2 genome sequences (1825 genomes available in GISAID on 30 March 2020) and showed that a protocol shared by the WHO for COVID-19 diagnostic used an oligonucleotide that could be ineffective at detecting up to 14% of the virus variants in circulation at the time and detected in 24 different countries [4]. This report strengthened the need to optimize the oligonucleotides used in COVID-19 diagnostics. At that point, the structure of the genetic population of SARS-CoV-2 was only starting to be defined. The dynamic nomenclature proposed for SARS-CoV-2 lineages [5] represented an extremely useful framework to identify the most spread genetic variants of the virus (lineages). Presently, despite the likely possibility that SARS-CoV-2 will continue to evolve (adapting to human host populations and/or in response to vaccination or other selective pressures), more than half a million genome sequences (526,401 available in GISAID on 15 March 2021) collected during more than one year of global transmission provided additional support for the rational design of the oligonucleotide to be used in RT-qPCR assays. This revealed extremely relevant as different prevalent lineages, such as B.1.1.7 (VUI-202012/01), have mutations known to cause target amplification failure in some COVID-19 molecular diagnostics methods [6,7,8,9,10]. The widely spread deletion of amino acids H69 and V70 of the Spike protein is one of the mutations with higher potential to interfere with methods designed from the reference genome to target this region [11].

The RT-qPCR oligonucleotides (primers and probes) for SARS-CoV-2 detection should be designed to bind regions of the viral genome that: (i) are highly conserved across the genetic diversity of the SARS-CoV-2 population, and (ii) are different in other viruses of the *Coronaviridae* family. In addition, the selected oligonucleotide sequences must respect the conditions necessary to be efficiently used in a qPCR reaction, such as adequate G/C content and low propensity to form secondary structures, among others. Here, this work aims at developing methods that account for these principles. We report a one-step multiplex RT-qPCR method (OminSARS2) for the fluorescent probe-based detection of three SARS-CoV-2 gene fragments that is specific and sensitive when detecting viral isolates from the most frequent SARS-CoV-2 lineages.

## 2. Materials and Methods

### 2.1. Primers and Probe Design

The sequence of our primer and probe designs are shown in Table 1. The primers and probes were designed with Geneious^®^ 7.0.6 (Auckland, New Zealand) to detect three different SARS-CoV-2 genes (ORF1ab, S and E) based on the Primer3 2.3.4 (http://primer3.sourceforge.net (accessed on 3 April 2020), considering the optimal length of the primer (25–27 bp), amplicon size (110–150 bp), predicted melting temperature in the 55–59 °C range, GC content 40–60% and absence of sequences that could lead to primer secondary structures (Table 1). The binding sites of the oligonucleotides in the reference SARS-CoV-2 genome and representative genomes of the 9 lineages currently designated as “Variants of Concern” and “Variants of Interest” are represented in Supplementary Figure S1. The human RNP gene was used as internal control to confirm, among others, the efficiency of sample collection, RNA extraction and absence of PCR inhibitors. Primers and probe nucleotide sequences for the detection of the human RNP gene were previously designed [12]. Primers and probes were purchased from Eurogentec (Seraing, Belgium). The specificity of these primer and probe sequences were further tested by using BLAST and the NCBI sequence databases.

To rapidly and more affordably quantify the three SARS-CoV-2 genes and the internal control gene in nasopharyngeal swabs, we combined four single RT-qPCR, amplifying the SARS-CoV-2 ORF1ab, S and E genes and the RNP (internal control), in a one-tube reaction. For this, the probes targeting the ORF1ab, S, E and RNP were differentially 5′-end labeled with FAM, ROX, HEX and CY5 reporter dyes. Black Hole Quencher^®^ (BHQ^®^) dye labeled at the 3′ end of the three SARS-CoV-2 probes and the BBQ for the human gene probe. The ORF1ab gene of SARS-CoV-2 is detected quantitatively by FAM channel, the S gene of SARS-CoV-2 detected quantitatively by ROX channel, the E gene of SARS-CoV-2 detected quantitatively by VIC channel, and the internal control (human RNP gene) detected by CY5 channel.

### 2.2. Patients Samples

Samples were collected from individuals by healthcare professionals using combined nose and/or throat swabbing and processed for diagnostic testing at national laboratories designated by the Portuguese government to proceed with SARS-CoV-2 molecular diagnostic. Specifically, for this work, excess samples from the diagnostic laboratories from UM/ICVS (http://www.icvs.uminho.pt/services-resources/covid19-diagnostic (accessed on 15 July 2021)) and Hospital of Braga were used to validate the in-house multiplex assays. A variety of swabs and viral transport media were used. In each case, swabs samples were kept at −80 °C until processed.

### 2.3. Nucleic Acid Extraction and RT-PCR Reaction Protocol

RNA was extracted from 200 µL of the nasopharyngeal swabs samples by NZY Viral RNA Isolation kit, MB40701 (©2020 NZYTech, Lda, Lisbon, Portugal) or through the semi-automated magnetic-particle processor (KingFisher Flex Purification System; Thermo Fisher Scientific, Waltham, Massachusetts, USA) according to the manufacturer instructions. The samples virus inactivation step, from the RNA extraction procedure, was conducted at BSL-2 laboratory. The RT-qPCR reactions were targeted for a final volume of 30 µL including 10 µL of target RNA and the remaining volume of SensiFAST™ Probe No-ROX One-Step Kit (™BioLine Meridian Bioscience, Memphis, TN, USA) or NZYSupreme One-Step RT-qPCR Probe Master Mix, MB414 (©2020 NZYTech, Lda, Lisbon, Portugal) and the oligonucleotides at a final concentration of 333 nM each SARS-CoV-2 primer, 84 nM each SARS-CoV-2 probe, 267 nM internal control primer and 67 nM internal control probe. The reactions were incubated at 45 °C for 10 min (reverse transcription), followed by 95 °C for 2 min (inactivation reverse transcriptase/polymerase activation) and 45 cycles of 95 °C for 5 s (denaturation), and 58 °C for 30 s (annealing/extension with signal acquisition). RT-qPCR assays were performed on a QuantStudio™ 6 Pro (Applied Biosystem by Thermo Fisher Scientific).

### 2.4. SARS-CoV-2 Genome Sequencing and Lineage Typing

Selected RNA samples were used to perform amplicon sequencing of SARS-CoV-2 following the ARTIC protocol v.3 [13]. This protocol is based on PCR amplification of the virus using tiled, multiplexed primers (ARTIC nCoV-2019 V3 Panel, IDT, CA, USA). Sequencing libraries were constructed with DNA Prep (M) Tagmentation kit (Illumina, CA, USA) using ¼ of the recommended volume and on average 125 ng of DNA input. QCs (Qubit and TapeStation) were performed in both PCR products and libraries. Sequencing was conducted in two different runs in an Illumina MiniSeq instrument (high output kit, PE150 reads) at the sequencing facility of the University of Vigo, Spain. The average sequencing depth was 1922.43X.

Reads were aligned to the reference MN908947.3 from Wu-Han using BWA-mem [14] and were then trimmed with iVar [15]. We evaluated the quality of the aligned trimmed reads using Picard v2.21.8 [16]. SAMtools depth v1.10 [17] was used to calculate the sequencing coverage along the genome. To build consensus sequences we used iVar consensus, indicating a minimum VAF threshold of 0.5. We then assigned them to a SARS-CoV-2 clade with Nextclade (https://clades.nextstrain.org (accessed on 6 July 2021)) and to a SARS-CoV-2 PANGO lineage [5] with Pangolin [18].

### 2.5. Statistical Analysis

The OmniSARS2 assay limit of detection (LoD) with 95% confidence level was determined using a commercially available RNA standard reference, EDX SARS-CoV-2 Standard (SKU: COV019, BioRad Inc., Hercules, CA, USA) with fourteen data points, each with fifteen replicate reactions and calculated using probit regression (dose–response analysis) with the software MedCalc^®^ v20.011. The EDX SARS-CoV-2 Standard contains synthetic RNA transcripts of SARS CoV-2 E, N, ORF1ab, RdRP and S genes, each quantitated at 200,000 copies/mL and human genomic DNA at 75,000 copies/mL. Fourteen serial ½ dilutions of the EDX SARS-CoV-2 Standard were performed up to a dilution concentration of 24 copies/mL of the SARS-CoV-2 genes. The linearity and efficiency for the quadruplex qRT-PCR were analyzed by Design and Analysis Software, Version: 2.4.3 (©2020 Thermo Fisher Scientific, Waltham, MA, USA) including linear regression and absolute quantification analysis. Spearman correlation coefficient of OmniSARS2 and two commercial kits (FOSUN and TaqPath) were measured using the average cycle quantification (Cq) values of the different SARS-CoV-2 genes present in each method, with matching clinical sample. Correlation coefficient, plots and significance were performed using GraphPad Prism 7 v7.04 (San Diego, CA, USA).

## 3. Results

### 3.1. In Silico Design of Oligonucleotide Sequences

**Figure 1:**
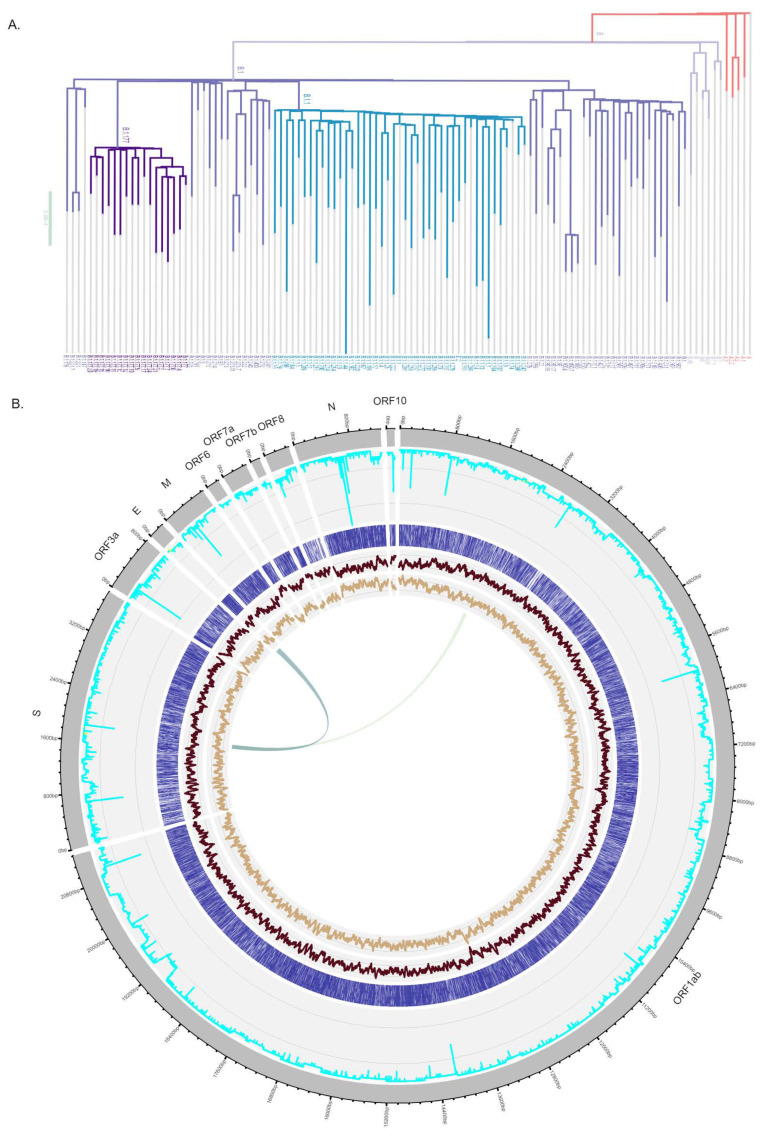
Genetic diversity of SARS-CoV-2. (**A**) Maximum likelihood phylogeny of SARS-CoV-2 genomes including one representative sequences from the 116 most common lineages globally sampled in the GISAID database on 15 March 2021. Lineage A and descendants were colored in red, and purple was used for lineage B and descendent. Within B, the B.1.1 and B.1.177 were colored in blue and dark purple, respectively. (**B**) Circular visualization of SARS-CoV-2 genome showing the genes in grey in the outer ring. Other tracks contain (from the outside in): the sequence identity line plot (cyan) across 116 genomes from the most common SARS-CoV-2 lineages; a bar plot indicating in blue the residues that are identical when comparing SARS-CoV-2 vs. SARS-CoV; line plots showing the AT (dark brown) and GC (light brown) contents of the SARS-CoV-2 genomes. Connecting ribbons in the center of the plot indicate the regions where the three sets of oligonucleotides from the reported assay were designed. NC_045512.2 was the reference genome.

To maximize the sensitivity and specificity of the method the design of the one-step multiplex RT-qPCR assay was based on the combination of a comparative genomics approach and a widely validated primer design algorithm [19]. To identify regions of difference between SARS-CoV-2 and the other *Coronaviridae* we performed pair-wise alignments of the reference genomes of SARS-CoV-2 and the genomes from four other betacoronavirus (HKU1, MERS-CoV, OC43, SARS-CoV) as well as two alphacoronavirus (229E and NL63). As expected, the largest identity (79.1%) was found between SARS-CoV (NC_004718.3) and SARS-CoV-2 (NC_045512.2). This allowed identifying the most unique regions of SARS-CoV-2 genome (Figure 1A) across the *Coronaviridae* family that are also the most suitable to be used in molecular detection methods that were SARS-CoV-2 specific. Given the increased disclosure of SARS-CoV-2 genetic variability, we also focused on the identification of the regions of the SARS-CoV-2 genomes that were more conserved within the most common lineages of the virus. To sample this diversity, the 526,401 available genomes (GISAID on 15 March 2021) were considered to randomly select one high-quality genome from each different lineage with more than 500 sequences in the database, leading to the selection of 116 genomes (Figure 1A). These genomes were aligned allowing the identification of conserved regions in all SARS-CoV-2 genes (Figure 1B) that could be used for the design of primers that equally detect the different lineages without target failure.

The sequences from SARS-CoV-2 genome that were absent or variable in other *Coronaviridae* and highly conserved across the most common SARS-CoV-2 lineages were used as templates to design a list of oligonucleotide sequences and to calculate the number of possible primer–template mismatches for all the designed oligonucleotides that could affect the polymerization efficiency resulting in biased RT-PCR results or reaction failure. The selection of the oligonucleotide sequences also considered the optimal length of the primer (25–27 bp), amplicon size (110–150 bp), predicted melting temperature in the 55–59 °C range, GC content 40–60%, and absence of sequences that could lead to primer secondary structures (Table 1). Overall, the designed oligonucleotide had the potential to be highly sensitive and specific for the detection of SARS-CoV-2.

### 3.2. Wet-Lab Determination of the Analytical Sensitivity and Specificity

**Figure 2:**
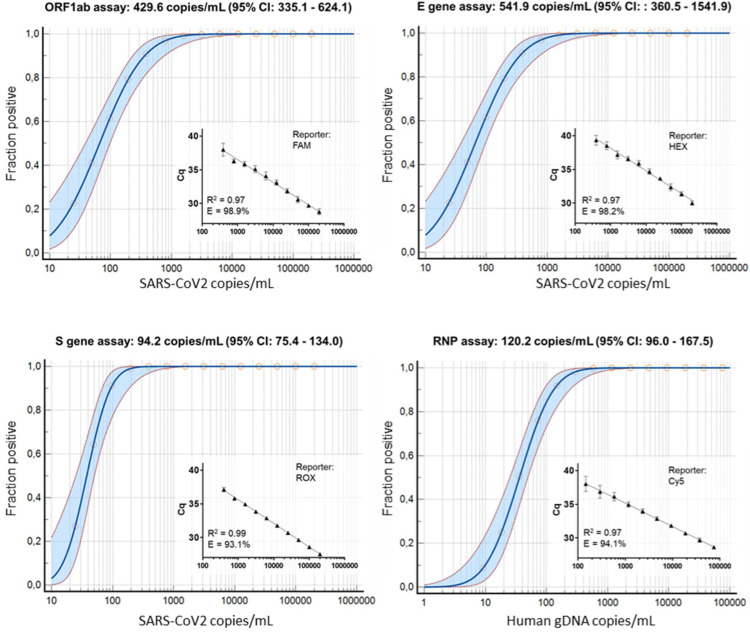
Determination of limits of detection based on the standard reference EDX SARS-CoV-2 for the three SARS CoV-2 genes (ORF1ab, S and E) and the human RNP gene. The *x*-axis shows nucleic acid copies per milliliter. The *y*-axis shows positive results in all reactions performed. Red circles represent fourteen experimental data points, each resulting from fifteen replicate testing. The fourteen data points were generated by serial 1:2 dilution of the standard reference EDX SARS-CoV-2 ranging from 200,000 copies/mL up to 24.4 copies/mL of SARS-CoV-2 synthetic RNA and 75,000 copies up to 9.2 copies/mL from human genomic DNA. Technical limits of detection are given in the panel headings. The blue inner line is a probit curve (dose–response analysis). The outer red lines are 95% probability confidence interval (CI). Inner graphs are standard curves generated from the mean quantification cycle (Cq) values (± SD for three independent assays each with three technical replicates) obtained against the copy number quantification of standard reference EDX SARS-CoV-2. The qPCR efficiency, coefficient of determination (R^2^) and the fluorophore tagged at the 5′ end of the probe (reporter) are also displayed in the inner graphs. RNP primers and probe nucleotide sequences previously designed [12]. ORF, open reading frame; E, envelope; S, spike; RNP, ribonuclease P; SARS-CoV-2, Severe Acute Respiratory Syndrome Coronavirus 2.

To determine the analytical sensitivity of OmniSARS2, we used a standard commercial reference containing synthetic RNA transcripts of SARS CoV-2 E, N, ORF1ab, RdRP and S genes and human genomic DNA to determine the limit of detection (LoD) with 95% confidence level. Fourteen different SARS-CoV2 RNA concentrations were tested ranging from 200,000 copies/mL up to 24 copies/mL, each subjected to fifteen replicate testing in order to determine stochastic detection frequencies at each assay’s sensitivity end point (Figure 2). The LoD of ORF1ab, E, S and RNP assays, ran in the multiplex reaction, revealed highly sensitive, with best results for S gene with 94.2 copies/mL at 95% detection probability (~1 copy per total reaction volume) and the least sensitivity for E gene with 541.9 copies/mL (~5 copies per total reaction volume). We have performed a standard curve, using serial 1:2 dilution of the EDX SARS-CoV-2 standard reference, to assess the performance of qPCR multiplex assay by estimating its efficiency. For the four assays (ORF1ab, E, S and RNP), each containing a different reporter dye, efficiency was detected above 90% which is considered the threshold for a properly designed assay (Figure 2).

Complementing the in-silico observation of no cross-reactivity with other human coronavirus, we used samples of known upper respiratory viruses, including betacoronavirus (OC43) and alphacoronavirus (229E and NL63), to evaluate the analytical specificity of the lab-developed multiplex RT-qPCR assay. All test results were found to be highly specific for SARS-CoV-2, with no cross-reactivity observed with other upper respiratory viruses (Supplementary Table S2). Overall, our results show that the developed multiplex assay is specific and can detect SARS-CoV-2 with high sensitivity.

### 3.3. Clinical Validation of the Multiplex RT-qPCR Assay

To validate OmniSARS2, 101 nasopharyngeal swabbing samples from individuals routinely tested for COVID-19 were used. The selected samples were previously diagnosed for SARS-CoV-2 at two different diagnostic laboratories using different commercial RT-PCR Kits. At UM-ICVS laboratories, 70 clinical samples, collected in September 2020 were diagnosed with FOSUN SARS-CoV-2 RT-qPCR Kit (Fosun Pharma, Xangai, China) comprising of 35 negatives and 35 positives for SARS-CoV-2. At the laboratories from Hospital of Braga, a set of 32 patient samples were collected in February 2021 and diagnosed as positive for SARS-CoV-2, using the Applied Biosystems TaqPath COVID-19 kit (Thermo Fisher Scientific, Waltham, Massachusetts, USA).

**Figure 3:**
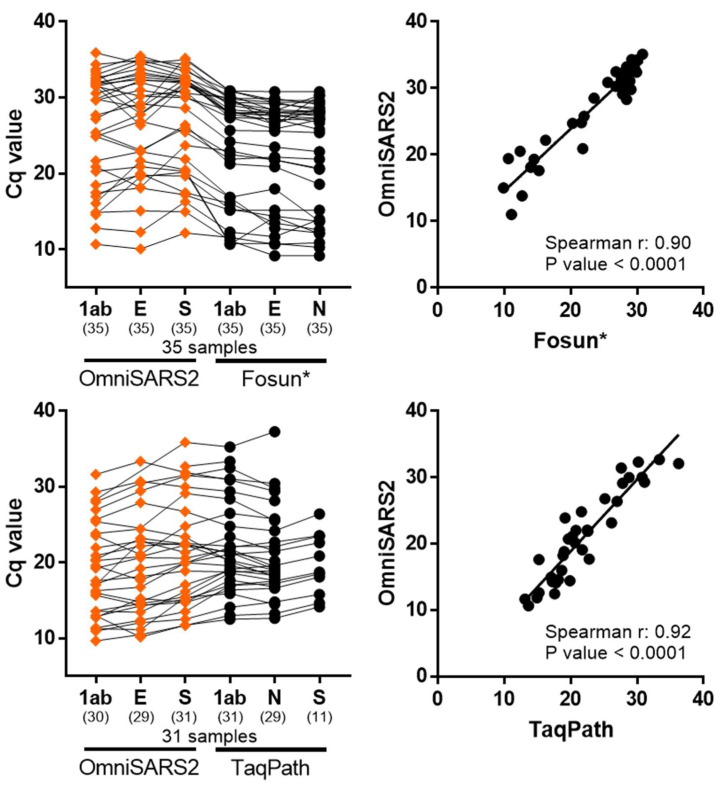
OmniSARS2 comparison with two commercial kits; Fosun and TaqPath. SARS-CoV-2 detection with OmniSARS2 was concordant to the Fosun and TaqPath commercial assays, correctly identifying all positive samples. Left graphs, represent clinical samples tested with the different methods. Connection lines between symbols represent matched clinical samples. The sensitivity of each of the three OmniSARS2 probes revealed high when compared with the TaqPath kit (detecting the N, Orf1ab, and S genes) which demonstrated miss-signal in 65% (detected 11 out of 31 samples) of the samples tested for the S probe. Additionally, see Supplementary Materials describing Cq values and result interpretation. Right graphs plot correlation of the 3 genes signal average between OmniSARS2 and the commercial kits. *, Fosun run method protocol set data collection and cycle record after the first 5 cycle runs, demanding interpretation caution when direct comparing the Cq values with the OmniSARS2 and TaqPath data; Cq, cycle quantification; 1ab, ORF1ab; E, envelope; N, nucleocapsid; S, spike; SARS-CoV-2, Severe Acute Respiratory Syndrome Coronavirus 2.

Both commercial kits and OmniSARS2 showed 100% positive agreement for high, medium, and low SARS-CoV-2 viral concentrations (Figure 3). However, some tested clinical cases from the diagnostic laboratories of Hospital of Braga, where cycle quantification (Cq) values were above 30, did not present signal in all three targeted viral genes when comparing OmniSARS2 and the TaqPath assay; nevertheless, this did not affect the overall qualitative interpretation of results. Noteworthy in the set of samples from Hospital of Braga, the sensitivity of each of the three probes of OmniSARS2 revealed high, with signal detected for targeted S gene present in 31 out of 31 samples, while TaqPath kit demonstrated extensive S gene dropout missing detection in 65% (detected 11 out of 31 samples) of the samples with the S gene probe (Figure 3).

### 3.4. Sensitivity Validation with Patient Samples

To validate how well OmniSARS2 detected the presence of different SARS-CoV-2 lineages we randomly selected a set of 174 SARS-CoV-2 positive nasopharyngeal samples collected in Braga to be sequenced. This allowed the identification of samples from 20 different SARS-CoV-2 lineages (Table 2). These included frequent and widely spread lineages such as B.1, B.1.1, B.1.177 or B.1.1.7 and rarer lineages.

**Figure 4:**
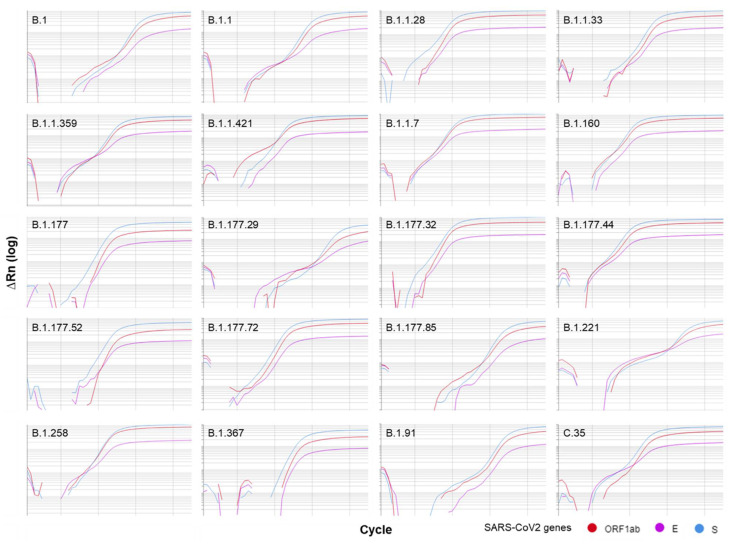
OmnisSARS2 detects different SARS-CoV-2 lineages. Representative qRT-PCR amplification plots showing the successful detection of the E, ORF1ab and S viral genes in nasopharyngeal samples from individuals infected with SARS-CoV-2 viruses from 20 different viral lineages.

The detection of SARS-CoV-2 lineage using OmniSARS2 was successful in all samples for the three targeted viral genes (Figure 4). These samples included viral lineages harboring several mutations in the genes ORF1ab, S and E when compared with the isolates obtained in the beginning of the COVID-19 epidemic. Our results showed detection of the three viral genes in all samples, supporting the absence of mismatches that interfere with the amplification.

## 4. Discussion

Having the most sensitive and specific diagnostic methods possible is of paramount importance in tracking and controlling any infectious disease. In COVID-19, several laboratories participated in the laudable efforts for the rapid development and open sharing of the first protocols and oligonucleotide sequences for SARS-CoV-2 detection. These were widely disseminated by the World Health Organization [20] and were critical for efficiently building the international capacity to diagnose COVID-19 since January 2020. The RT-qPCR is presently recognized as the reference method allowing the highest specificity and sensitivity. However, SARS-CoV-2 has accumulated several polymorphisms during the COVID-19 pandemic. Some of these spontaneous and recurrent mutations, such as the substitution GGG->AAC in the N gene [4] or the deletion of amino acids 69 and 70 in S protein [21], can interfere with the thermal stability of the primer/probe–template duplex, potentially compromising the effective SARS-CoV-2 detection.

In this study, we developed a one-step multiplex RT-qPCR assay (OmniSARS2) for the simultaneous detection of three SARS-CoV-2 genes and one human control gene based on the combination of a comparative genomics approach and a gold standard primer design protocol. We considered the SARS-CoV-2 genome evolution and selected unique and conserved sequences of the SARS-CoV-2 at the ORF1ab, E and S gene, maximizing the specificity of the assay. Moreover, we added to the multiplex assay, an endogenous human control gene (RNP), as a reference to monitor the nucleic acids extraction and sampling quality, avoiding false-negative results [22].

Our developed assay revealed a high analytical sensitivity, with the best results being obtained for S gene assay with 94.2 copies/mL at 95% detection probability (~1 copy per total reaction volume) and the least sensitivity for E gene with 541.9 copies/mL (~5 copies per total reaction volume). These results are well below the average sensitivity of a reference panel of 117 SARS-CoV-2 molecular in vitro diagnostic (IVD) assays for which analytical performance was tested [23]. Albeit different methods and equipment were used, our results suggest OmniSARS might be more sensitive than most of the previously tested [23] commercially available RT-qPCR methods for SARS-CoV-2 detection.

In addition, as proof of sensitivity, our results on 101 clinical samples compared with two widely used commercial kits presented 100% agreement with the qualitative results, with the highest variable Cq values related with the differential sample viral load and not between the methods. The negative patients demonstrate the same result for the different methods, guaranteeing the test diagnostic accuracy. Nonetheless, it will be relevant to further test the method in the future to access its clinical specificity using a larger and broader panel of clinical samples from other respiratory diseases.

We have also challenged the analytical specificity of the developed assay, with other human *Coronaviridae* of known upper respiratory viruses and with several SARS-CoV-2 lineages. The results revealed high specificity with no mismatch nucleotides detected in the whole-genome sequencing of clinical samples, under the primers and probes binding site, thus assuring a high specific assay not compromised by the genetic diversity of SARS-CoV-2 lineages.

The emergence and rapid spread of novel lineages carrying several mutations when compared with the reference SARS-CoV-2 genome, such as B.1.1.7, reinforced the relevance of designing RT-qPCR methods with oligonucleotides targeting regions that are highly conserved across the genetic diversity of the SARS-CoV-2. These genomic regions are less likely to accumulate mutations that could interfere with the results. The main advantage of RT-qPCR for the diagnostic of COVID-19 when compared with other methods such as the ones based on the detection of specific immune responses is its sensitivity [24]. The increased sensitivity of RT-qPCR allows it to detect infections even in patients with low viral loads that are typical of early stages of infection. Detecting infections at early stages has obvious advantages in preventing transmission. In addition to infection stage, other factors such as viral lineage, host characteristics, variability in sample collection or viral RNA degradation in the collected sample might lead to biological samples that are “sub-optimal” or more challenging for diagnostic. Particularly for these samples, relying on the detection of more than one viral gene is of relevance to avoid false negatives.

In this work we openly share a highly sensitive and specific COVID-19 diagnostic method based on the simultaneous detection of three viral genes, promoting the adoption of updated and reliable diagnostics that will be key for controlling the COVID-19 pandemic and effectively mitigating the emergence of transmission foci in the post-pandemic period.

## Figures and Tables

**Table 1:** Design of the one-step quadruplex qRT-PCR assays for the detection of three SARS-CoV-2 genes and internal human control gene.

Target Gene	Oligo Name	Sequence (5’-3’)	Amplicon Size
SARS-CoV-2	1ab1917F1	AGAGTTTCTTAGAGACGGTTGG	111 bp
ORF1ab	1ab1977P1	FAM-TGTCGGTGGACAAATTGTCACCTGT-BHQ	
	1ab2003R1	TGAACACTCTCCTTAATTTCCTTTG	
SARS-CoV-2	S1703F1	ACATTGCTGACACTACTGATGC	119 bp
Spike (S)	S1725P1	ROX-TGTCCGTGATCCACAGACACTTGAG-BHQ	
	S1797R1	CTGGTTAGAAGTATTTGTTCCTGGT	
SARS-CoV-2	E22F1	AGACAGGTACGTTAATAGTTAATAGCG	144 bp
Envelope (E)	E84P1	HEX-AGTTACACTAGCCATCCTTACTGCGC	
	E143R1	AAGAAGGTTTTACAAGACTCACGT-BHQ	
^1^ Human	RNP_F	AGATTTGGACCTGCGAGCG	
Ribonuclease P	RNP_R	GAGCGGCTGTCTCCACAAGT	65 bp
	RNP_P	Cy5-TTCTGACCTGAAGGCTCTGCGCG-BBQ	

^1^ Primers and probes previously designed [13].

**Table 2:** Lineage in 174 SARS-CoV-2 positive nasopharyngeal samples randomly selected for sequencing.

Lineage ^1^	Sample (*n*)	Sampling Dates	Common Countries#
B.1	13	16 April 2020–22 September 2020	USA 46.0%, GBR 10.0%, DEU 5.0%, ESP 3.0%, IND 3.0%
B.1.1	88	03 April 2020–16 November 2020	GBR 33.0%, USA 15.0%, JPN 8.0%, DEU 4.0%, RUS 3.0%
B.1.1.28	12	25 August 2020–13 January 2021	BRA 60.0%, PHL 17.0%, USA 8.0%, URY 5.0%, JPN 2.0%
B.1.1.33	1	09 November 2020	BRA 81.0%, USA 5.0%, CHL 4.0%, URY 1.0%, ARG 1.0%
B.1.1.359	1	04 November 2020	GHA 70.0%, DNK 15.0%, BFA 7.0%, USA 4.0%, TGO 4.0%
B.1.1.421	2	17 June 2020	PRT 74.0%, GBR 15.0%, USA 6.0%, RUS 3.0%, CHE 3.0%
B.1.1.7	2	13 January 2021	GBR 30.0%, USA 20.0%, DEU 11.0%, DNK 6.0%, SWE 6.0%
B.1.160	1	20 November 2020	DNK 16.0%, FRA 16.0%, CHE 11.0%, GBR 9.0%, DEU 8.0%
B.1.177	5	18 May 2020–13 January 2021	GBR 62.0%, ESP 11.0%, DEU 5.0%, CHE 4.0%, ITA 4.0%
B.1.177.29	1	11 January 2021	ESP 50.0%, ITA 31.0%, PRT 12.0%, GBR 6.0%
B.1.177.32	5	29 December 2020	ESP 39.0%, PRT 38.0%, CHE 9.0%, LUX 3.0%, FRA 3.0%
B.1.177.44	5	01 October 2020–20 November 2020	CHE 79.0%, DEU 5.0%, ITA 4.0%, GBR 3.0%, NLD 2.0%
B.1.177.52	9	03 November 2020–13 January 2021	PRT 37.0%, GBR 26.0%, DEU 18.0%, LUX 4.0%, NLD 3.0%
B.1.177.72	13	22 October 2020–13 January 2021	PRT 73.0%, FRA 8.0%, CHE 7.0%, LUX 5.0%, ESP 4.0%
B.1.177.85	1	08 January 2021	PRT 61.0%, CHE 20.0%, LUX 13.0%, FRA 4.0%, GBR 1.0%
B.1.221	1	11 January 2021	NLD 20.0%, DEU 17.0%, DNK 14.0%, BEL 11.0%, SWE 10.0%
B.1.258	1	13 January 2021	DEU 20.0%, GBR 18.0%, DNK 16.0%, SWE 8.0%, CHE 6.0%
B.1.367	4	01 September 2020	GBR 32.0%, FRA 17.0%, CHE 11.0%, NOR 10.0%, DNK 7.0%
B.1.91	8	14 April 2020–04 November 2020	PRT 64.0%, GBR 13.0%, BRA 5.0%, NZL 5.0%, ITA 3.0%
C.35 (alias of B.1.1.1.35)	1	28 July 2020	DEU 19.0%, GBR 17.0%, CHE 12.0%, DNK 11.0%, GRC 7.0%

**^1^** Lineage obtained with pangolin 2.4.2, pangoLEARN version 19/05/2021, pango version v1.2.6. # Data obtained from PANGO lineages (https://cov-lineages.org/ (accessed on 18 June 2021)) indicating countries were SARS-CoV-2 clinical isolates from the given lineage were frequently sequenced. ISO 3166-1 alpha-3 country codes were used.

## Supplementary Materials

The following are available online at https://www.mdpi.com/article/10.3390/biomedicines9101314/s1, Figure S1: Graphical representation of the binding site of the OmnisSARS2 oligonucleotides in a reference SARS-CoV-2 genome (NC 045512) and representative genomes of SARS-CoV-2 lineages that were more frequent or emerging in 2021. Table S1: List of microorganisms tested for cross-reactivity by in silico analysis. Table S2: Cross-Reactivity: Microorganisms analyzed by wet lab testing. Table S3: Number of clinical samples for each SARS-CoV-2 lineage and positivity of detection using OmniSARS2.
