# Supplementary material for: OmniSARS2: A Highly Sensitive and Specific RT-qPCR-Based COVID-19 Diagnostic Method Designed to Withstand SARS-CoV-2 Lineage Evolution
Source: Biomedicines. 2021 Sep 26;9(10):1314. doi: 10.3390/biomedicines9101314 (PMC8533632; doi:10.3390/biomedicines9101314)
Supplement: Supplementary file 1 [file biomedicines-09-01314-s001.zip › biomedicines-1321944-supplementary tables.pdf]

Supplementary data

Supplementary Table S1: List of microorganisms tested for cross-reactivity by *in silico* analysis.

**Microorganism**	
Human coronavirus 229E	Influenza A virus, H1N1
Human Coronavirus OC43	Influenza A virus, H3N2
Human Coronavirus HKU1	Influenza B virus
Human Coronavirus NL63	Human parainfluenza virus 1
SARS Coronavirus	Human parainfluenza virus 2
MERS Coronavirus	Human parainfluenza virus 3
Human adenovirus 1	Human respiratory syncytial virus A
Human adenovirus 2	Human respiratory syncytial virus B
Human adenovirus 3	Human metapneumovirus
Human adenovirus 4	*Chlamydia pneumoniae*
Human adenovirus 5	*Haemophilus influenzae-B*
Human adenovirus 7	*Legionella pneumophila*
Human adenovirus 55	*Mycobacterium tuberculosis*
Enterovirus D68	*Streptococcus pneumonia-19*
Human enterovirus 71	*Streptococcus pyogenes*
Human rhinovirus 1A	*Bordetella pertussis*
Human rhinovirus 14	*Mycoplasma pneumoniae*
Human rhinovirus 57	*Candida albicans*
Human rhinovirus 1B	*Pneumocystis jirovecii (PJP)*
Human rhinovirus C	*Staphylococcus salivarius*

Supplementary Table S2: Cross-Reactivity: Microorganisms analyzed by Wet Testing with OminSARS2 method§

Virus strain	Sample type	OmniSARS2				
		Ct - SARS-CoV-2 genes			Ct - Human gene	Results
		ORF1ab	E	S	RNP	
*Human coronavirus 229E (NR-52726)	Genomic RNA isolated from 200μL cell lysate and supernatant from human lung fibroblast cells infected with HCoV, 229E	UND	UND	UND	25.7	ND
*Human coronavirus OC43 (NR-52725)	Genomic RNA isolated from 200μL cell lysate and supernatant from human ileocecal colorectal adenocarcinoma cells infected with HCoV, OC43	UND	UND	UND	22.2	ND
*Human coronavirus NL63 (NR44105)	Genomic RNA isolated from a cell lysate and supernatant from monkey kidney cells (LLC-MK2) infected with HCoV, NL63.	UND	UND	UND	38.3	ND

*: The following reagent was obtained through BEI Resources, NIAID, NIH. UND: undetermined; ND: Not Detected.

§: OmniSARS assay, has been used, since November 2020, to diagnose more than 5000 clinical samples at the diagnostic laboratory of University of Minho. Accordingly, the OmniSARS assay has been evaluated by external quality assessment program (AEQ), promoted by the National Health Institute Doctor Ricardo Jorge (INSA), through the National Program for External Quality Assessment (PNAEQ) and by the 2020 World Health Organization (WHO) international Proficiency Testing Provider (PTP) for the detection of SARS-CoV-2 by PCR, evaluating the laboratory performance and proposing recommendations (lab code: PNAEQ_610_1/2021 and lab code: WHO/30422_2/2021). These external evaluations included the testing of the sensitivity and specificity of the method with clinical samples from other respiratory disease. Moreover, OmniSARS assay was used in a national program for monitoring the flu virus and other respiratory disorders and showed no cross-reactivity in clinical samples infected with other virus such as Picornavirus and Syncytial Respiratory virus.

Supplementary Table S3: Number of clinical samples for each SARS-CoV-2 lineage and positivity of detection using OmniSARS2.

**Lineage**	**Number of clinical samples**	**SARS-CoV-2 Positivity detected with OMNISARS2 (n, %)**
**B.1**	13	13, 100%
**B.1.1**	88	88, 100%
**B.1.1.28**	12	12, 100%
**B.1.1.33**	1	1, 100%
**B.1.1.359**	1	1, 100%

**B.1.1.421**	2	2, 100%
**B.1.1.7**	2	2, 100%
**B.1.160**	1	1, 100%
**B.1.177**	5	5, 100%
**B.1.177.29**	1	1, 100%
**B.1.177.32**	5	5, 100%
**B.1.177.44**	5	5, 100%
**B.1.177.52**	9	9, 100%
**B.1.177.72**	13	13, 100%
**B.1.177.85**	1	1, 100%
**B.1.221**	1	1, 100%
**B.1.258**	1	1, 100%
**B.1.367**	4	4, 100%
**B.1.91**	8	8, 100%
**C.35**	1	1, 100%
