# Supplementary material for: OmniSARS2: A Highly Sensitive and Specific RT-qPCR-Based COVID-19 Diagnostic Method Designed to Withstand SARS-CoV-2 Lineage Evolution
Source: Biomedicines. 2021 Sep 26;9(10):1314. doi: 10.3390/biomedicines9101314 (PMC8533632; doi:10.3390/biomedicines9101314)
Supplement: Supplementary file 1 [file SupplementaryFigureS1.pdf › SupplementaryFigureS1.pdf]

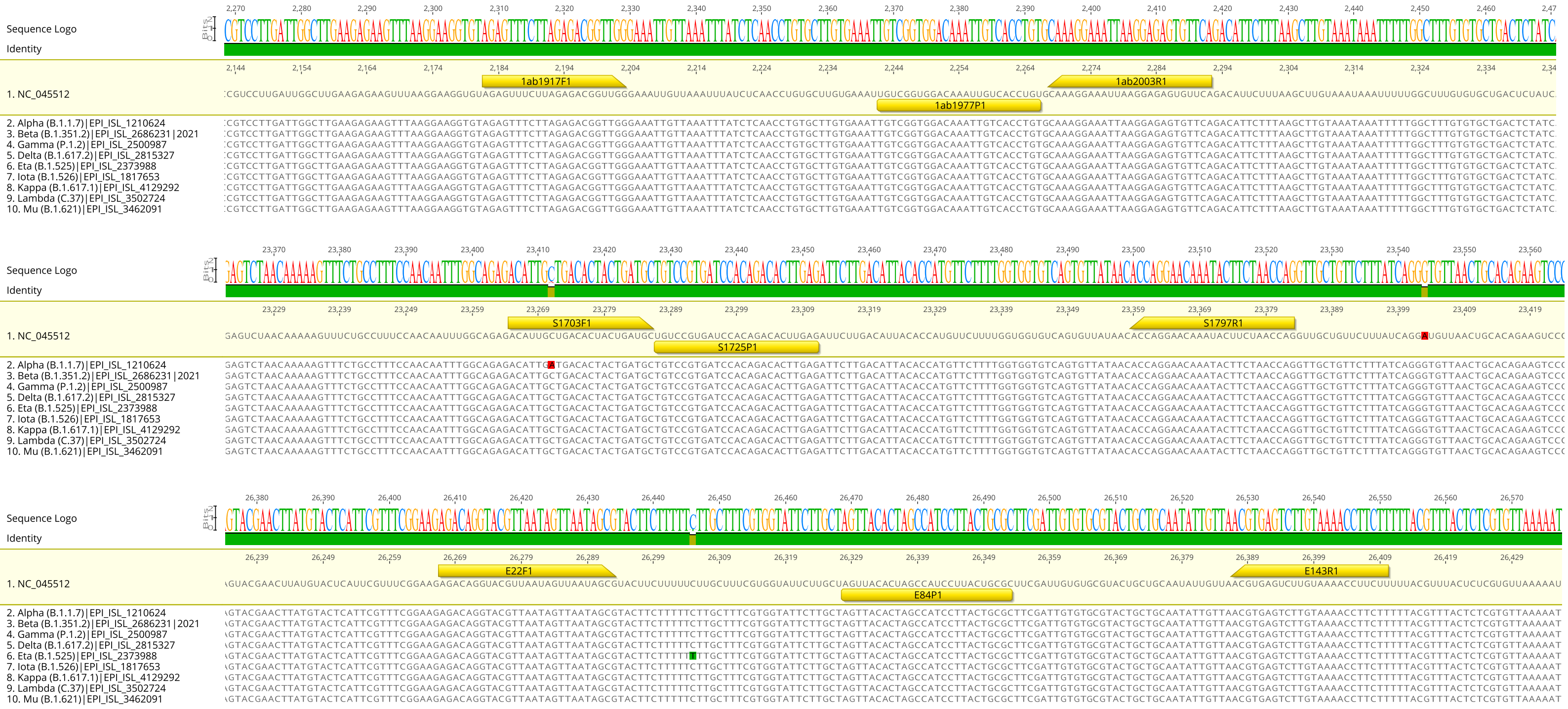
Sequence Logo
Identity
1. NC_045512
1ab1917F1
1ab2003R1
1ab1977P1
S1703F1
S1797R1
S1725P1
E22F1
E143R1
E84P1
2. Alpha (B.1.1.7)|EPI_ISL_1210624
3. Beta (B.1.351.2)|EPI_ISL_2686231|2021
4. Gamma (P.1.2)|EPI_ISL_2500987
5. Delta (B.1.617.2)|EPI_ISL_2815327
6. Eta (B.1.525)|EPI_ISL_2373988
7. Iota (B.1.526)|EPI_ISL_1817653
8. Kappa (B.1.617.1)|EPI_ISL_4129292
9. Lambda (C.37)|EPI_ISL_3502724
10. Mu (B.1.621)|EPI_ISL_3462091
